# Lactic Acid Bacteria from Northern Thai (Lanna) Fermented Foods: A Promising Source of Probiotics with Applications in Synbiotic Formulation

**DOI:** 10.3390/foods14020244

**Published:** 2025-01-14

**Authors:** Nittiya Suwannasom, Achiraya Siriphap, Ornampai Japa, Chonthida Thephinlap, Chutamas Thepmalee, Krissana Khoothiam

**Affiliations:** 1 Division of Biochemistry, School of Medical Sciences, University of Phayao, Phayao 56000, Thailand; nittiya.su@up.ac.th (N.S.); chonthida.th@up.ac.th (C.T.); chutamas.th@up.ac.th (C.T.); 2 Division of Microbiology, School of Medical Sciences, University of Phayao, Phayao 56000, Thailand; achiraya.si@up.ac.th (A.S.); ornampai.ja@up.ac.th (O.J.)

**Keywords:** lactic acid bacteria (LAB), northern Thai (Lanna) fermented foods, probiotics, synbiotic

## Abstract

Northern Thai culture offers a rich variety of traditional fermented foods beneficial for gastrointestinal health. In this study, we characterized lactic acid bacteria (LAB) from various indigenous fermented foods as potential probiotic candidates and determined their properties for application in commercial synbiotic formulation. Five isolates demonstrating high tolerance to low pH (2.0) and 0.3% bile salts were collected and characterized. These included three strains of *Lactiplantibacillus plantarum* isolated from nham (NB1, NP2, and NP11) and two strains of *Limosilactobacillus fermentum* isolated from pla-som (PS4 and PS7). All the selected LAB isolates exhibited γ-hemolytic activity, strong antimicrobial activity, and high resistance to gastric and duodenal digestion conditions. Among the LAB isolates, *L. plantarum* NB1 demonstrated the highest capacity for adhesion to Caco-2 cells, auto-aggregation, and antioxidant activity, differing significantly (*p* < 0.05) from the other isolates. Furthermore, the NB1 strain exhibited preferential growth in the presence of commercial prebiotics (fructooligosaccharide, lactose, and inulin) and good survival after lyophilization, which is a desirable characteristic for a powdered ingredient. Therefore, the NB1 strain is a suitable probiotic candidate for applications in synbiotic formulation or as a functional food ingredient.

## 1. Introduction

Probiotics have emerged as a subject of increasing interest and research in the field of health and nutrition over the past decade [1]. When consumed in sufficient quantities, these living microorganisms offer numerous health advantages to the host. Probiotics maintain and improve the balance of the intestinal microbiota, leading to improved digestion and enhanced immunity. They also help to reduce cholesterol levels and increase resistance to various intestinal infections [2]. To be effective, probiotics must possess the ability to withstand the challenging conditions of the gastrointestinal (GI) tract, such as low pH and the presence of bile salts [3]. Additionally, they must adhere to the intestinal mucosa to successfully colonize the gut and exert their beneficial health effects on the host, which are essential characteristics of effective probiotics [2]. Probiotics, primarily from the genus of *Lactobacillus*, *Leuconostoc*, *Pediococcus*, and *Bifidobacterium*, are mostly found in various food products, including fermented dairy and non-dairy items [4,5].

Northern Thai culture, also known as Lanna, is rich in a diverse array of traditional fermented foods, each with its own unique history, preparation methods, and microbial composition. Lanna fermented foods predominantly include non-dairy fermented foods, which utilize a variety of ingredients like vegetables, fish, and other meats. These fermented delicacies include nham, which is commonly made from ground pork but can also be made from buffalo meat; both versions are prepared as fermented sausages [6], and pla-som, a fermented freshwater fish product. Among the fermented vegetable foods, phak-gard-dong and naw-mai-dong are well-known products made from mustard greens and bamboo shoots, respectively [7].

Unlike many modern food products, traditional Lanna ferments rely on naturally occurring microflora to drive the fermentation process, resulting in a diverse array of flavors, aromas, and potential health benefits [8]. Several previous studies have shed light on the remarkable microbial diversity, especially of lactic acid bacteria (LAB), found within these traditional ferments. For instance, *Lactobacillus* sp., *Pediococcus pentosaceus*, and *Streptococcus* sp. are predominantly found in nham [9], while *Pediococcus cerevisiae*, *Lactobacillus brevis*, and *Bacillus* sp. are mostly present in pla-som [10]. *Leuconostoc mesenteroides*, *P. cerevisiae*, *Lactobacillus plantarum*, *L. brevis*, and *Lactobacillus fermentum* are dominantly recovered from naw-mai-dong and phak-gard-dong [7], suggesting that they may offer more than just food preservation benefits. These bacteria have been shown to potentially have probiotic effects as well.

Synbiotics combine prebiotics and probiotics that work together synergistically, enhancing host health by improving the survivability and productivity of beneficial microorganisms in the GI tract [11]. Fructooligosaccharide (FOS), lactose, and inulin have been commonly recognized as prebiotics and are widely used in food industries worldwide [12]. Hence, the objective of this study was to obtain and characterize potentially probiotic LAB from indigenous fermented foods specific to local geographic areas, especially in the Phayao province in Northern Thailand. The aim is to eventually produce a commercial synbiotic formulation, as well as functional food ingredients.

## 2. Materials and Methods

### 2.1. Sample Collection

Thirty-five samples of Lanna fermented foods, such as naw-mai-dong, nham, phak-gard-dong, and pla-som, were collected from five marketplaces in the Phayao province in Northern Thailand during June 2023. All collected samples were stored at room temperature until analysis.

### 2.2. Isolation of LAB

The samples (10 g) were homogenized in peptone water (90 mL), and serial dilutions were prepared. These dilutions were then inoculated onto de Man Rogosa and Sharpe (MRS) agar (HiMedia, Mumbai, India) supplement with bromocresol green. The plates were subsequently incubated at 37 °C for a period of 24 to 48 h. The colonies of acid-producing bacteria were identified and selected on MRS agar by the presence of a yellow zone surrounding each colony. The LAB isolates were analyzed for their morphological and biological characteristics using the method established by Lee et al. [3]. Only Gram-positive, catalase-negative, and oxidase-positive strains were required. The LAB strains were stored at −20 °C until use.

### 2.3. Acid and Bile Salt Tolerance Test

All LAB isolates were tested to determine their tolerance to low pH according to the method described by Argyri et al. [13], with minor modifications. The cells of LAB strains were harvested, washed, and then suspended to a concentration of approximately 10^8^ CFU/mL in MRS broth adjusted to pH 2.0 using hydrochloric acid, with cells suspended in MRS broth (pH 7.2) serving as controls. Acid tolerance was determined by assessing viability, enumerated as log CFU/mL.

The bile salt resistance of LAB strains was assessed according to the method previously described by Gu et al. [14], with a few modifications. The LAB cultures were inoculated into two sets of MRS broth: one containing 0.3% bile salt and the other without bile salt, along with a control. The samples were then incubated. All results were reported as log CFU/mL, and the survival rate was determined using the following formula:

Survival (%) = [final (log CFU/mL)/control (log CFU/mL)] × 100(1)

### 2.4. Identification of LAB Isolates Using 16S Ribosomal DNA Analysis

The LAB strains demonstrating high tolerance to acid and bile salt were identified by 16S rDNA sequencing analysis. Genomic DNA from these isolates was extracted using the boiling method. Primers 27F (5′-AGAGTTTGATCCTGGCTCAG-3′) and 1525R (5′-AAGGAGGTGWTCCARCC-3′) were utilized to amplify the 16S rRNA gene. The PCR products were then submitted for sequencing to a service provider. The 16S rRNA gene sequence was utilized to identify the most closely related sequences by employing a Basic Local Alignment Search Tool (BLAST) analysis, which was accessible through the NCBI GenBank databases. Multiple sequence alignments were conducted for each dataset. A phylogenetic tree was constructed using the neighbor-joining method in MEGA11: Molecular Evolutionary Genetics Analysis version 11 [15] and visualized with FigTree 1.4.4 software.

### 2.5. Antimicrobial Activity

The antibacterial activity of the cell-free culture supernatant (CFCS) of the LAB isolates was evaluated against four pathogens: *Escherichia coli* TISTR 073, *Salmonella enteritidis* DMST 15676, *Bacillus cereus* TISTR 1813, and *Staphylococcus aureus* TISTR 746 using the agar diffusion method, as previously described by Unban et al. [16]. Additionally, *L. plantarum* TISTR 543 served as the reference probiotic control. The pathogens were cultured in Mueller–Hinton broth (MHB) (HiMedia, Mumbai, India). The bacterial cultures were inoculated onto the surface of Mueller–Hinton agar plates using cotton swabs. Subsequently, paper disks containing 20 µL of neutralized CFCS adjusted to pH 7.0, as well as non-neutralized CFCS (not adjusted for pH), were applied to the surface of the plates. Following incubation, the diameters of the growth inhibition zones were measured.

### 2.6. Hemolytic Activity Test

The hemolytic activity of selected LAB was investigated as described by Haghshenas et al. [17]. To determine hemolytic activity, the LAB strain was inoculated onto blood agar. Three types of hemolysis were observed on the blood agar: the presence of clear zones around the colonies was indicative of β-hemolysis, while greenish-gray zones surrounding the colonies signified α-hemolysis. Conversely, the absence of clear zones around the colonies suggested γ-hemolysis.

### 2.7. Antibiotic Susceptibility

The LAB strains’ antibiotic sensitivity was assessed using the disk diffusion technique. Seven antibiotic disks were used, including chloramphenicol, ciprofloxacin, clindamycin, erythromycin, penicillin G, streptomycin, and vancomycin. Fifty microliters of overnight culture of LAB isolates was spread on MRS agar, and the antibiotic disks were positioned on the surface of the agar plates. The plates were then incubated, and the results were evaluated by measuring the diameter of the zone of inhibitory growth and interpreted as sensitive (S), intermediate (I), or resistant (R) according to the CLSI [18].

### 2.8. In Vitro GI Digestion Conditions

The LAB isolates were assessed for probiotic properties, including resistance to gastrointestinal conditions, as described by Sriphannam et al. [19], with minor modifications. The cells of the LAB isolates and the reference strain (*L. plantarum*) were harvested, washed, and then resuspended in an electrolyte solution. To determine viability at time 0, the solution was aliquoted, serially diluted in normal saline solution, and inoculated onto MRS agar plates. To mimic the dilution and potential hydrolysis reaction that would occur in the oral cavity, the cell solution was combined with an equal volume of an electrolyte solution containing lysozyme. The cell suspension and electrolyte solution containing lysozyme were then incubated at 37 °C for 5 min. After this incubation, the mixture was diluted at a ratio of 3:5 with an artificial gastric solution. This gastric solution was composed of 0.3% pepsin in an electrolyte solution, with the pH adjusted to 2.5. The diluted mixture was then incubated for 1 h at 37 °C, after which a sample was collected to quantify viability on an MRS medium. The remaining volume from the previous step was further diluted at a ratio of 3:5 using an artificial duodenal secretion to simulate the conditions in the duodenum. This diluted mixture was then incubated at 37 °C. Samples were collected at the 2 h and 3 h time points during this incubation period. Viability was examined, and the survival rate was calculated.

### 2.9. Determination of Cell Surface Hydrophobicity

The hydrophobicity of LAB isolates was investigated as described by Garcia-Gonzalez et al. [20], with minor modifications. The cells of the LAB strains were harvested, washed, and then resuspended to obtain an optical density (OD) of 1.0 at 600 nm (Initial OD). The mixture was homogenized for 5 min after adding an equal volume of chloroform. Following a 2 h incubation period, the optical density of the aqueous phase was determined at 600 nm (Final OD). The hydrophobicity was calculated using the following formula:

Hydrophobicity (%) = [(Initial OD − Final OD)/Initial OD] × 100(2)

### 2.10. Cell Adhesion Assay

The colon adenocarcinoma cell line Caco-2 was employed to investigate the adhesion ability of LAB isolates, as previously described by Piatek et al. [21], with a few modifications. Caco-2 cells were cultured under controlled conditions in Dulbecco’s Modified Eagle Medium (DMEM) (HyClone, Logan, UT, USA). Once the Caco-2 monolayer reached 90% confluence, the cells were sub-cultured in 96-well plates and incubated for 15 days. The selected LAB isolates were cultured in MRS broth for 24 h, followed by centrifugation to harvest the cells. The cells were then resuspended in DMEM. The Caco-2 cells were washed with PBS, and bacterial cell suspensions were transferred to the plates. The plates were incubated for 4 h before being washed with PBS to eliminate non-adherent bacteria. Subsequently, the monolayers were mixed with Triton X-100 (0.5%) (Sigma-Aldrich, Darmstadt, Germany) to remove the Caco-2 monolayer and the adherent bacteria from the inoculated wells. The adherent bacteria were cultivated on MRS agar. To assess the percentage of adhesion, the number of adherent bacteria was compared to the total number of bacterial cells.

### 2.11. DPPH Scavenging Ability

The 2,2-diphenyl-1-picrylhydrazyl (DPPH) free radical scavenging ability of selected LAB isolates and their CFCS was evaluated as described by Chen et al. [22], with minor modifications. DPPH solution was freshly prepared and then added to bacterial cells and CFCS. The reaction mixture was kept at room temperature in the dark for 30 min. The scavenging capacity of DPPH was determined at 517 nm. DPPH solution and a mixture of bacterial cells/CFCS and methanol were used as the control and blank, respectively. The potential scavenging ability was calculated using the following formula:

DPPH scavenging ability (%) = [(Sample OD − Blank OD)/Control OD] × 100(3)

### 2.12. Auto- and Co-Agglutination Assay

The auto- and co-agglutination capacities of LAB isolates were examined as previously described by Del Re et al. [23], with minor modifications. The cells of the LAB isolates were harvested, washed, resuspended in PBS buffer (HiMedia, Mumbai, India ), and mixed. Next, the cell mixture was measured at 600 nm (Initial OD). The mixture was incubated at 37 °C for 4 h, after which the absorbance of the upper mixture was measured at 600 nm (Final OD). The percentage of auto-agglutination was determined using the following formula:

Auto-agglutination (%) = [(Initial OD − Final OD)/Initial OD] × 100

To investigate co-agglutination, five selected LAB strains were grown in MRS broth, and four pathogens (*E. coli*, *S. enteritidis*, *B. cereus*, and *S. aureus*) were grown in MHB. Bacterial suspensions were prepared as described in the auto-agglutination method. The LAB isolate solution was mixed with the pathogen and incubated at 37 °C for 4 h. The cell mixture was measured for absorbance OD 600 nm at time 0 (Initial OD) and 4 h (Final OD). The percentage of co-agglutination was determined using the following formula:

Co-agglutination (%) = [(Initial OD − Final OD)/Initial OD] × 100(4)

### 2.13. Prebiotic Utilization Ability

The capacity of LAB strains to utilize prebiotics was investigated and demonstrated as a prebiotic score (PS), as described by Unban et al. [16] with minor modifications. FOS, lactose, and inulin were used as the prebiotic substrates for LAB strain cultivation. The cells of the LAB isolates were harvested, washed, and then resuspended in PBS buffer to achieve the desired concentration. Subsequently, the bacterial suspension was added to modified MRS broth with 10 g/L of each prebiotic (FOS, lactose, and inulin) as the sole carbohydrate source and then incubated. Modified MRS medium with glucose (10 g/L) as the carbohydrate substrate served as the positive control. Prebiotic utilization was evaluated by detecting the absorbance OD 620 nm of LAB isolates grown with each prebiotic substrate (Pre OD) or glucose (Glu OD). Bacterial growth on the carbohydrate substrate glucose was defined as 100%. The PS was determined using the following formula:

PS (%) = (Pre OD/Glu OD) × 100(5)

### 2.14. Lyophilization and Survival Test

The cells of the LAB were harvested, washed, and then resuspended in a lyoprotector solution to obtain a concentration of 10^8^ CFU. Next, the suspension was frozen for at least 4 h before undergoing the freeze-drying procedure. The freeze-drying process was performed using a lyophilizer. Bacterial viability was assessed both before and after the freeze-drying process. The survival rate after freeze-drying was calculated as a percentage using the following formula:

Survival rate (%) = (N/N0) × 100 (6)

In this equation, N represents the number of colonies per gram in the dried sample, and N0 represents the number of colonies per gram in the sample solution prior to freeze-drying, according to the method used by Juárez Tomás et al. [24].

### 2.15. Statistical Analysis

All experiments were conducted in triplicate, and the data were expressed as mean ± standard deviation (mean ± SD). The results were analyzed using one-way analysis of variance (ANOVA) in GraphPad Prism (version 5.00 for Windows) using a *p*-value < 0.05 for the determination of significance.

## 3. Results and Discussion

### 3.1. Isolation of LAB

Fifty-one colonies of acid-producing bacteria were isolated from four different types of Northern Thai fermented foods on MRS medium. Based on the biochemical and morphological characteristics of LAB, such as being Gram-positive, catalase-negative, and oxidase-positive, fourteen LAB isolates were collected from different fermented samples (Table 1), including three isolates from naw-mai-dong (bamboo shoot pickle), six from nham (fermented meat sausage), three from pla-som (fermented fish product), and two from phak-gard-dong (pickled mustard greens). Several studies have revealed that LAB are the predominant microorganisms in fermented food products [2,9]. In this study, nham (a fermented meat product) was found to be the dominant source of LAB, consistent with research by Rungrassamee et al. [25], which showed that nham is an important source of *Lactobacillus* genus members such as *L. sakei*, *L. delbrueckii*, *L. plantarum*, and *L. fermentum*. Additionally, other studies noted that although the genus *Lactobacillus* comprises the majority of LAB found in nham, the genera *Pediococcus* and *Streptococcus* are also present [6]. All LAB strains were further evaluated for their resistance properties under acidic and bile salt conditions.

### 3.2. Tolerance to Low pH and Bile Salt

Tolerance to low acidity and bile salts is essential for probiotics to survive in the GI tract [2]. The pH of the stomach varies from 1.5–2 to 3.5–5 after food is digested [26]; therefore, in vitro tests usually use a pH range of 2–3 to select probiotic strains that can survive in gastric pH conditions [3]. In this study, a pH of 2.0 was used to eliminate isolates that may have potential probiotic properties but are unable to survive in low pH conditions. Fourteen LAB strains were examined for their acid tolerance after incubation for 3 h under pH 2.0. All the LAB strains showed high survival rates (>80%) in low-acid conditions (Table 1). Isolate NP2 from nham (fermented pork-meat sausage) exhibited the highest tolerance to acidic conditions, with a survival rate of 97.84%. Our results agree with previous research reporting that the *Lactobacillaceae* family exhibits high viability in acidic environments, as concluded by Pakwan et al. regarding the bacterial compositions of indigenous Lanna foods [9]. Therefore, all isolates were further evaluated for their ability to tolerate bile salts.

Tolerance to bile salts is essential for probiotics because the human GI tract contains high levels of bile salts, approximately 500–700 mL/day (a bile salt concentration of 0.3%), which can damage bacterial cells [27]. Most previous studies used 0.3% (*w*/*v*) bile salts as the recommended concentration for screening tolerant strains [3,28]. Therefore, 0.3% bile salt was used in this study. The results showed that five out of the fourteen isolates were tolerant to 0.3% bile salt, with a survival rate exceeding 80%, while the remaining isolates exhibited tolerance in the range of 63.94% to 76.53% (Table 1). This finding is in agreement with a study by Gu et al. [29] and supported by Maldonado et al. [30], which shows that bile tolerance is not necessarily species-specific but can be a strain-specific feature. Since tolerance to low acidity and bile salts is a critical factor for selecting effective probiotics, we assessed this combined tolerance in all five isolates (NB1, NP2, NP11, PS4, and PS7). Consequently, these isolates were selected for further species-level identification.

### 3.3. Identification of LAB

Five selected LAB isolates were identified using 16S rRNA gene sequence analysis. The similarity of the DNA sequences was determined using the NCBI-BLAST database, and the results are shown in Table 2. In this study, three different species were classified: *Lactiplantibacillus plantarum* (formerly *Lactobacillus plantarum*, isolates NB1 from fermented buffalo meat and NP2 and NP11 from fermented pork meat), and *Limosilactobacillus fermentum* (formerly *Lactobacillus fermentum*, isolates PS4 and PS7 from fermented fish). 

**Figure 1:**
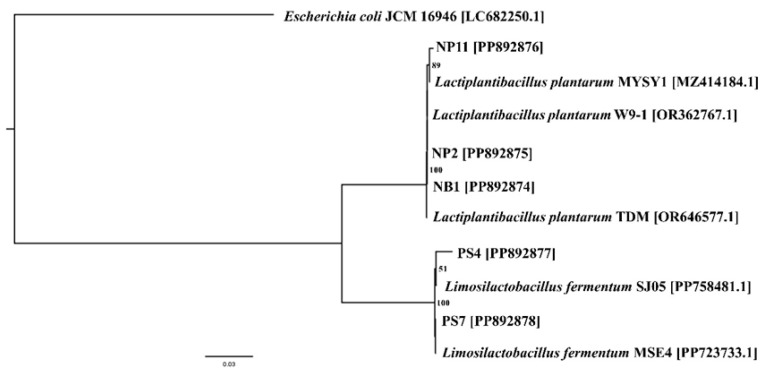
Phylogenetic tree based on 16S rRNA gene sequencing analysis of the five LAB isolates and other closely related species.

The phylogenetic tree analysis revealed the species closest to the five selected LAB isolates (Figure 1). Our results are consistent with previous studies which reported that the dominant LAB species found in fermented meat products, including fermented pork and fermented freshwater fish, were *L. plantarum*, *L. fermentum*, *Lactobacillus sakei*, *Lactobacillus delbrueckii*, *Lactococcus lactis,* and *Lactococcus garvieae* [9,25].

### 3.4. Antimicrobial Activity

The LAB strains were assessed for antimicrobial activity using both neutralized and non-neutralized CFCS against foodborne pathogens such as *E. coli*, *S. enteritidis*, *S. aureus*, and *B. cereus*. The results showed that the non-neutralized CFCS obtained from the five LAB isolates, as well as the reference probiotic strain *L. plantarum,* exhibited varying degrees of inhibitory effects against the tested pathogens (Table 3). Our results are consistent with other investigations reporting that the inhibitory effects of non-neutralized CFCS are likely due to the production of organic acids, hydrogen peroxide, or other low-molecular-weight metabolites by the LAB species [31]. In contrast, the neutralized CFCS did not show any inhibitory activity, suggesting that the antimicrobial properties of these LAB strains are not due to bacteriocin-like compounds [32]. The inhibition zones produced by the non-neutralized CFCS of the LAB isolates ranged from 8.0 to 13.67 mm in diameter. The isolate *L. plantarum* NB1 showed the highest inhibitory activity against *S. aureus*, with an inhibition zone of 12.67 ± 0.58 mm, significantly different (*p* < 0.05) from that with *E. coli* and *B. cereus* but not *S. enteritidis* (*p* > 0.05). The results are consistent with another study that showed that *L. plantarum* species are highly effective at inhibiting the growth of Gram-positive foodborne pathogens, especially *S. aureus* [33]. This efficacy is greater than that against Gram-negative bacteria such as *E. coli*, *Salmonella typhimurium*, and *Listeria monocytogenes* [34], likely due to the outer membrane of Gram-negative bacteria acting as a protective barrier. However, most of the non-neutralized CFCS obtained from LAB isolates exhibited comparable inhibitory effects against the four different pathogens when compared to the probiotic reference strains. This finding is supported by numerous previous studies, including Haghshenas et al., who demonstrated the antimicrobial activity of *L. plantarum* against various pathogens [17], and Sankar et al., who reported similar inhibitory effects from bacteriocin produced by *L. plantarum* isolated from cow milk [32]. These results suggest that the selected LAB isolates, particularly those from the genus *Lactobacillus*, possess potent antimicrobial properties that effectively inhibit the growth of a wide variety of foodborne pathogens, encompassing both Gram-positive and Gram-negative bacteria [19].

### 3.5. Survival in Gastric and Duodenal Digestion Conditions

**Figure 2:**
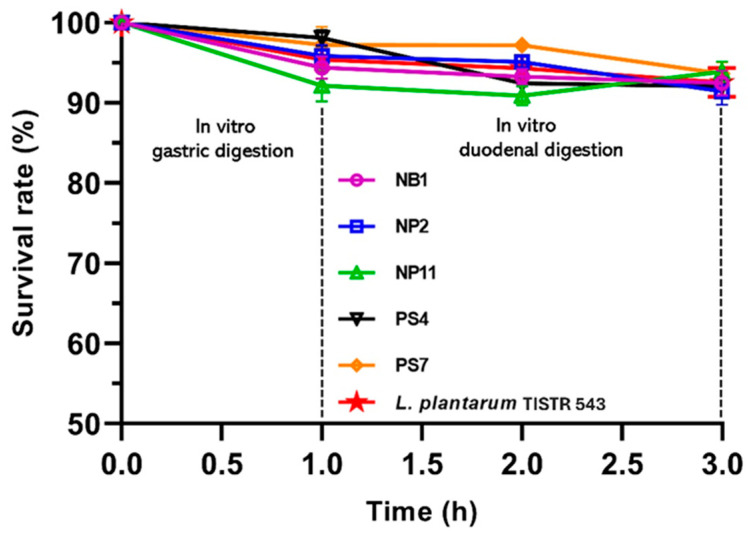
Survival of *L. plantarum* NB1, *L. plantarum* NP2, *L. plantarum* NP11, *L. fermentum* PS4, and *L. fermentum* PS7 under in vitro gastric and duodenal digestion conditions. *L. plantarum* TISTR 543 was used as the probiotic reference strain.

The effects of in vitro GI conditions on the survival of LAB strains and the reference probiotic strain were evaluated and are presented in Figure 2. The results showed that the survival rates of each LAB isolate at the end of treatment with artificial duodenal digestion fluid ranged from 91.48% to 93.90%, which were statistically comparable to the viability of the *L. plantarum* probiotic strain (92.58%). All five selected LAB strains demonstrated good tolerance and achieved more than 90% survival after being subjected to in vitro GI conditions. Our results are similar to previous research, which reported the high tolerance of LAB isolates to simulated GI conditions, with most strains exhibiting over 80% survival rates after 3 h of incubation in simulated gastric juice [5]. The enhanced survival of the selected LAB isolates, particularly *L. plantarum* species (NB1, NP2, and NP11), under in vitro GI conditions can be attributed to the presence of meso-diaminopimelic acid (mDAP) in their cell walls. The mDAP peptidoglycan structure is known to provide better tolerance against stressful environments, which can translate to greater survival ability in simulated GI conditions [20].

### 3.6. Hemolytic Activity and Antibiotic Susceptibility

The selected LAB isolates exhibited γ-hemolytic activity (Table 4), which indicates that they did not display any α- or β-hemolytic properties. This is a desirable characteristic for probiotic strains, as the absence of hemolytic activity suggests a low risk of pathogenicity [13]. The antibiotic susceptibility testing of the LAB isolates revealed that they were resistant to ciprofloxacin and vancomycin (Table 4). This pattern of antibiotic resistance and susceptibility aligns with the findings of Gupta et al. [14] and Peres et al. [35], who also observed similar antibiotic resistance profiles in their LAB isolates. Notably, the data showed that *L. plantarum* NP11 exhibited further resistance to penicillin G, in addition to its resistance to ciprofloxacin and vancomycin. This enhanced antibiotic resistance profile of *L. plantarum* NP11 may be an advantageous trait, as it could provide this strain with increased survival and persistence in the GI environment, where antibiotic exposure is common. The observed antibiotic resistance patterns of the LAB isolates are consistent with the literature, which suggests that intrinsic antibiotic resistance is a common characteristic among *Lactobacillus* species [36].

### 3.7. Hydrophobicity

Cell surface hydrophobicity is an important characteristic of probiotic bacteria, as it is directly related to their ability to adhere to intestinal epithelial cells and compete with pathogens. Our results showed that the selected LAB strains exhibited a degree of hydrophobicity ranging from 43.63% to 66.28% towards chloroform (Table 4). This is consistent with previous studies that reported variations in hydrophobicity among the genus *Lactobacillus* (43–79%), as demonstrated by Todorov et al., who highlighted the beneficial properties of lactic acid bacteria isolated from smoked salmon [37]. Notably, *L. plantarum* NP11 exhibited remarkable hydrophobicity (66.28%) in chloroform compared to the others (*p* < 0.05). The high hydrophobicity of the LAB strains suggests their potential to interact with the mucosal cells of the GI tract, which could enhance their ability to exclude pathogens [38]. The variation in hydrophobicity among probiotic LAB strains is significant, as it reflects their individual adhesion capabilities [39].

### 3.8. Adhesion Capacity to Caco-2 Cells

Numerous studies have highlighted the relationship between cell surface hydrophobicity and the adhesion capacity of probiotic LAB strains in colonizing the gut [5,40]. The adhesion capacity to Caco-2 cells, commonly used as a model for intestinal epithelial cells, was determined for the five selected LAB isolates. The results demonstrated that the selected LAB isolates revealed varying degrees of adhesion capacity to Caco-2cells, which mostly correlated with their hydrophobicity (Table 4). *L. plantarum* NB1, which demonstrated the highest adhesion capacity (78.22%), also displayed significant hydrophobicity, at 53.10%. However, *L. fermentum* PS4, which had the lowest adhesion ability at 58.77%, exhibited moderate hydrophobicity (50.14%). Several investigations have demonstrated a strong correlation between hydrophobicity and adhesion capacity [41,42], a relationship also observed in this study. However, another study found no evidence to support this correlation [28]. The strain-specific variations in the adhesion capacity of the LAB isolates may be influenced by factors beyond hydrophobicity. The expression of specific adhesion proteins, including mucus-binding proteins, could play a crucial role in the adhesion process [43].

### 3.9. Auto-and Co-Agglutination Ability

The ability of the family *Lactobacillaceae* to aggregate can create a barrier, potentially preventing pathogenic strains from adhering to the GI tract [44]. The results of the current study showed that the auto-aggregation of the selected LAB strains ranged from 28.80% to 56.11%, with *L. plantarum* NB1 exhibiting a high auto-aggregation value of 56.11% (*p* < 0.05; Table 5). This is in accordance with the findings of Piwat et al. [45], who reported that the auto-aggregation ability of LAB strains, such as *Lactobacillus rhamnosus* and *L. plantarum*, can exceed 50% after 24 h of incubation. Regarding co-aggregation, the data showed that the levels of co-aggregation between the LAB strains and the tested pathogens (*E. coli*, *S. enteritidis*, *S. aureus*, and *B. cereus*) varied, ranging from 10.14% to 47.72% (Table 5). This is consistent with the values reported by Sophatha et al. [46], indicating that the co-aggregation of *Lactobacillus* strains with pathogens, such as *E. coli* and *Salmonella typhimurium*, can range from 21% to 66% after 24 h of incubation. In this study, the highest co-aggregation value was recorded between *L. plantarum* NB1 and *E. coli* strains (47.72%), which is similar to the finding of Janković et al. [47] that the isolate *L. plantarum* S1 demonstrated the highest co-aggregation with enterohaemorrhagic *E. coli*, achieving a co-aggregation rate of 41.5%. Our results imply that the auto-aggregation ability of LAB strains may be linked to their capacity to co-aggregate with pathogens, as both processes are believed to contribute to the exclusion and inhibition of pathogenic bacteria in the gut [48]. The strong auto-aggregation and co-aggregation properties of *L. plantarum* NB1 support this potential relationship, as this strain may have a higher ability to adhere to intestinal epithelial cells and displace or inhibit the adhesion of pathogens like *E. coli* [49]. The results suggest that the auto-aggregation and co-aggregation abilities are strain-specific, with varying values observed among the LAB isolates [47,49].

### 3.10. Free Radical Scavenging Activity

The antioxidant capacity of the five selected LAB isolates was investigated through DPPH radical scavenging activity. The CFCS of the five isolates exhibited DPPH scavenging activities ranging from 66.50% to 74.43% (Table 4). Particularly, *L. plantarum* NB1 showed the highest scavenging activity quite significantly at 74.43% (*p* < 0.05), followed by *L. plantarum* NP11 (72.74%), *L. fermentum* PS4 (70.72%), *L. plantarum* NP2 (69.58%), and *L. fermentum* PS7 (66.50%). A similar finding was demonstrated by Unban et al. [16], noting that the CFCSs of all the selected *Lactobacillus* isolates exhibited higher radical scavenging capacities than their intact cells. Antioxidant mechanisms, such as the production of antioxidases present in intact cells or antioxidant compounds like phenolic acids found in CFCS, may contribute to these differences in a strain-specific manner [50].

### 3.11. Prebiotic Utilization

**Figure 3:**
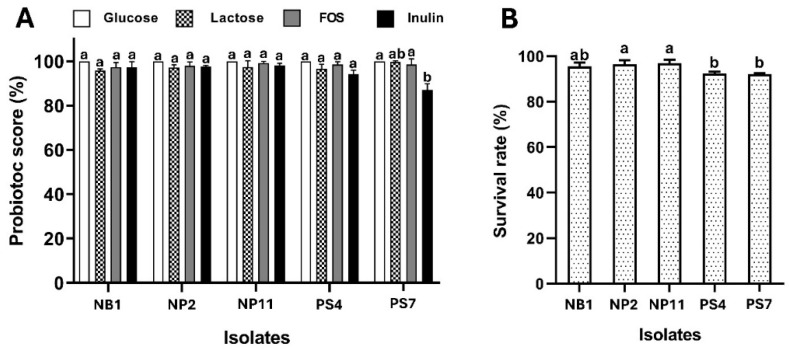
Probiotic scores (**A**) and survivability after lyophilization (**B**) of *L. plantarum* NB1, *L. plantarum* NP2, *L. plantarum* NP11, *L. fermentum* PS4, and *L. fermentum* PS7. Statistically significant differences (*p* < 0.05) between strains are indicated by different lowercase letters (a,b).

The five selected LAB isolates exhibited excellent growth in the presence of 1% (*w*/*v*) of the three prebiotic sugars of lactose, FOS, and inulin, as well as glucose, which was used as the positive control (Figure 3A). *L. plantarum* NB1, *L. plantarum* NP2, *L. plantarum* NP11, and *L. fermentum* PS4 exhibited the highest PS for cells grown with FOS (97.31%, 97.97%, 99.21%, and 98.45%, respectively), whereas *L. fermentum* PS7 exhibited a PS of 99.66% with lactose. These results contrast with previous reports that showed *Lactobacillus* species had higher growth rates with inulin than with other prebiotics [51]. This suggests strain-dependent prebiotic preferences and growth characteristics of the selected LAB strains, which can be useful in designing effective synbiotic formulations. Thus, further investigations are necessary to determine the specific interactions between prebiotics and probiotics, which is crucial for refining their commercial application.

### 3.12. Survival of LAB After Lyophilization

The survival values of the five selected LAB strains during the freeze-drying process were investigated and are presented in Figure 3B. *L. plantarum* NB1, *L. plantarum* NP2, *L. plantarum* NP11, *L. fermentum* PS4, and *L. fermentum* PS7 retained survival levels of 95.56%, 96.43%, 96.99%, 92.34%, and 92.12%, respectively, after the lyophilization process. These findings indicate much higher survival rates compared to those reported in previous studies, such as Jalali et al.’s [52], which recorded a maximum survival rate of *Lactobacillus* species of approximately 72–76%, and our prior research [53], which reported the viability of the reference probiotic *L. plantarum* after lyophilization as 73.74%. Our results demonstrate that the selected LAB strains exhibit excellent survival rates, tolerating inconvenient conditions during the freeze-drying process. This high viability makes them promising candidates for large-scale production and potential application in commercially available products.

## 4. Conclusions

This study reported the isolation and identification of LAB from various Northern Thai traditional fermented foods and evaluated their probiotic properties, including tolerance to low pH and bile salts, antimicrobial activity, and other characteristics essential for effective probiotics. Five selected LAB strains exhibited high survival rates under acidic conditions and tolerance to bile salts and showed strong antimicrobial activity. Among the LAB strains, *L. plantarum* NB1, isolated from fermented buffalo meat sausage, demonstrated good adhesion to Caco-2 cells, high levels of auto- and co-aggregation, and strong antioxidant activity. Additionally, *L. plantarum* NB1 had an excellent growth rate in the presence of commercial prebiotics and maintained a higher percentage of viability after lyophilization. Therefore, *L. plantarum* NB1 has strong potential as a probiotic candidate for further in vivo investigation as a functional food ingredient or as part of a synbiotic formulation. However, a limitation of this study is the small sample size, as it includes only 35 samples of Lanna fermented foods collected from five marketplaces in the studied area.

## Figures and Tables

**Table 1:** Origin of Northern Thai fermented food and survival rates of isolated LAB after 3 h of incubation at pH 2.0 and with 0.3% bile salt.

Isolates	Origin	Survival Rate (%)	
		pH 2.0	0.3% Bile Salt
BS-3	Naw-mai-dong (bamboo shoot pickle)	82.27 ± 1.65 ^f^	72.40 ± 2.33 ^d^
BS-5	Naw-mai-dong (bamboo shoot pickle)	83.42 ± 1.52 ^f^	72.63 ± 1.12 ^d^
BS-8	Naw-mai-dong (bamboo shoot pickle)	82.45 ± 1.27 ^f^	73.05 ± 2.42 ^d^
NB1	Nham (fermented buffalo meat sausage)	96.88 ± 1.14 ^ab^	88.64 ± 0.65 ^b^
NB4	Nham (fermented buffalo meat sausage)	93.12 ± 1.54 ^bc^	74.77 ± 1.40 ^d^
NP2	Nham (fermented pork sausage)	97.84 ± 0.53 ^a^	95.01 ± 1.39 ^a^
NP5	Nham (fermented pork sausage)	91.98 ± 1.93 ^cd^	72.31 ± 1.86 ^d^
NP9	Nham (fermented pork sausage)	92.12 ± 1.73 ^cd^	76.53 ± 1.49 ^d^
NP-11	Nham (fermented pork sausage)	88.63 ± 1.18 ^de^	97.44 ± 1.86 ^a^
PS4	Pla-som (fermented fish product)	89.15 ± 0.57 ^cd^	83.26 ± 2.22 ^c^
PS7	Pla-som (fermented fish product)	92.95 ± 1.60 ^bc^	83.83 ± 1.25 ^bc^
PS12	Pla-som (fermented fish product)	91.94 ± 1.40 ^cd^	71.84 ± 0.98 ^d^
PG2	Phak-gard-dong (pickled mustard greens)	83.08 ± 1.50 ^f^	63.94 ± 1.88 ^e^
PG3	Phak-gard-dong (pickled mustard greens)	84.52 ± 0.83 ^ef^	65.21 ± 1.82 ^e^

The values presented are the means ± standard deviations of three experiments. Different lowercase letters (a–f) within the same column indicate statistical significance at *p* < 0.05.

**Table 2:** Genotypic characterization of the five selected LAB strains.

Isolates	Species Identified	Similarity (%)	Sequence Length (bp)	Accession Number
NB1	*Lactiplantibacillus plantarum*	100	1097	PP892874
NP2	*Lactiplantibacillus plantarum*	99.45	1097	PP892875
NP11	*Lactiplantibacillus plantarum*	99.82	1096	PP892876
PS4	*Limosilactobacillus fermentum*	98.81	1096	PP892877
PS7	*Limosilactobacillus fermentum*	100	1101	PP892878

**Table 3:** The inhibitory effect of LAB isolates against pathogenic bacteria.

Isolates	Diameter of Inhibition Zone (mm)			
	*E. coli*	*S. enteritidis*	*S. aureus*	*B. cereus*
NB1	9.00 ± 0 ^ABc^	12.00 ± 1.73 ^Aab^	12.67 ± 0.58 ^Ba^	9.67 ± 0.58 ^BCbc^
NP2	8.00 ± 0 ^Bc^	10.00 ± 0 ^ABb^	12.00 ± 0 ^BCa^	12.67 ± 0.58 ^ABa^
NP11	8.00 ± 0 ^Bc^	9.67 ± 0.58 ^Bb^	9.000 ± 0 ^Cb^	11.00 ± 0 ^Ba^
PS4	8.33 ± 0.58 ^ABc^	10.67 ± 0.58 ^ABb^	13.67 ± 0.58 ^Ba^	13.33 ± 1.15 ^Aa^
PS7	8.33 ± 0.58 ^ABb^	9.00 ± 0 ^Bb^	13.67 ± 2.31 ^Ba^	9.00 ± 0 ^Cb^
*L. plantarum*	9.33 ± 0.58 ^Ab^	11.00 ± 0 ^ABb^	19.33 ± 1.15 ^Aa^	10.67 ± 0.58 ^BCb^

Values represent means ± standard deviations of triplicate. Different uppercase letters (A–C) in the same column indicate statistical significance (*p* < 0.05). Differences indicated by lowercase letters (a–c) within the same line are also significant (*p* < 0.05).

**Table 4:** Hemolytic activity, antimicrobial resistant profiles (AMR), hydrophobic ability, adhesion property, and antioxidant potential of the LAB isolates.

Isolates	Test				
	Hemolysis	AMR	Hydrophobicity (%)	Adhesion (%)	DPPH (%)
NB1	γ	CIP, VA	53.10 ± 2.71 ^b^	78.22 ± 0.81 ^a^	74.43 ± 1.79 ^a^
NP2	γ	CIP, VA	44.91 ± 2.85 ^c^	68.48 ± 0.85 ^c^	69.58 ± 0.54 ^bc^
NP11	γ	CIP, VA, P	66.28 ± 1.91 ^a^	73.70 ± 0.53 ^b^	72.74 ± 0.64 ^b^
PS4	γ	CIP, VA	50.14 ± 2.71 ^bc^	58.72 ± 0.58 ^d^	70.72 ± 1.77 ^b^
PS7	γ	CIP, VA	43.63 ± 2.93 ^c^	59.06 ± 1.10 ^d^	66.50 ± 2.08 ^c^

γ: no hemolytic activity, CIP: ciprofloxacin, P: penicillin G, VA: vancomycin. Values expressed as means ± standard deviations of triplicate. Different lowercase letters (a–d) in the same column represent statistical significance (*p* < 0.05).

**Table 5:** Auto-agglutination and co-agglutination abilities of the five selected LAB isolates.

Isolates	Auto-Agglutination (%)	Co-Agglutination (%)			
		*E. coli*	*S. enteritidis*	*S. aureus*	*B. cereus*
NB1	56.11 ± 1.15 ^a^	47.72 ± 0.29 ^a^	24.88 ± 1.73 ^a^	39.59 ± 1.64 ^a^	40.92 ± 1.16 ^a^
NP2	45.00 ± 1.06 ^c^	43.01 ± 0.83 ^b^	13.27 ± 2.77 ^bc^	38.52 ± 1.67 ^ab^	19.47 ± 1.28 ^b^
NP11	50.84 ± 1.41 ^b^	42.73 ± 1.19 ^b^	16.03 ± 0.98 ^b^	34.02 ± 1.50 ^b^	40.50 ± 2.23 ^a^
PS4	28.80 ± 1.07 ^e^	15.54 ± 3.78 ^d^	10.14 ± 1.00 ^bc^	16.29 ± 2.26 ^d^	20.00 ± 0.68 ^b^
PS7	32.17 ± 0.74 ^d^	27.02 ± 1.83 ^c^	17.98 ± 2.04 ^b^	24.13 ± 2.07 ^c^	18.05 ± 1.72 ^b^

The values represent means ± standard deviations of three determinations. Different lowercase letters (a–e) in the same column indicate significant differences (*p* < 0.05).

## Data Availability

The original contributions presented in this study are included in the article. Further inquiries can be directed to the corresponding author.
